# The First N,O-Chelated Diphenylboron-Based Fluorescent Probe for Peroxynitrite and Its Bioimaging Applications

**DOI:** 10.3390/bios14110515

**Published:** 2024-10-22

**Authors:** Xiaoping Ye, Longxuan Li, Hong Liu, Yuyu Fang, Xiaoya Liu

**Affiliations:** 1 Department of Ultrasound, The First Affiliated Hospital of Chongqing Medical University, Chongqing 400010, China; 2 School of Pharmacy, Chengdu University of Traditional Chinese Medicine, Chengdu 611137, China; 3 Department of Vascular Surgery, The First Affiliated Hospital of Chongqing Medical University, Chongqing 400010, China; 4 Department of Oncology, The First Affiliated Hospital of Chongqing Medical University, Chongqing 400010, China

**Keywords:** fluorescent probe, peroxynitrite, ROS, boron chelates, bioimaging

## Abstract

Peroxynitrite (ONOO^−^) is a reactive oxygen species (ROS) that takes part in the oxidation-reduction homeostasis while at the same time being responsible for activating numerous pathological pathways. Accordingly, monitoring the dynamic changes in ONOO^−^ concentration has attracted a great deal of attention, undoubtedly prompting the development of appropriate fluorescent chemosensors. Herein, we developed a novel N,O-chelated diphenylboron-based fluorescent probe (DPB) for ONOO^−^ featuring high selectivity, a quick response time (2.0 min), and a low detection limit (55 nM). DPB incorporates tetra-coordinated boron in the center of the fluorogenic core and a three-coordinated boron from the pinacolphenylboronate fragment, which acts as the recognition site for ONOO^−^. As confirmed by HR-MS and ^1^H NMR, the interaction of DPB with ONOO^−^ led to an oxidative cleavage of pinacolphenylboronate moiety to produce strongly emissive derivative DPB-OH. The fluorescence enhancement is likely a result of a substantial deactivation of non-radiative decay due to the replacement of the bulky pinacolphenylboronate moiety with a compact hydroxyl group. Importantly, DPB probe exhibits negligible cytotoxicity and favorable biocompatibility allowing for an efficient tracking of ONOO^−^ in living cells and zebrafish. Overall, the current study does not only represents the first N,O-chelated diphenylboron-based fluorescent probe for a specific analyte, but also serves as a guideline for designing more potent fluorescent probes based on the chemistry of boron chelates.

## 1. Introduction

The oxidation-reduction processes play a key role in many life functions, facilitating the maintenance of a healthy and steady physiological state [1]. The redox homeostasis preserves the balance between the physiological oxidants and the reducing agents to keep their concentrations in the appropriate range through the continuous signaling for their production and elimination [2]. On the other hand, excessive levels or high concentrations of oxidants can damage various biological molecules and cell components (e.g., proteins, nucleic acids, lipids, etc.), thereby leading to signal disruptions and oxidative damage, which may potentially induce severe diseases and premature aging [1,2]. Reactive oxygen species (ROS) are among the most important types of oxidants in living organisms, consisting of a set of oxygen-containing substances in the form of ions, molecules, and radicals [3]. ROS are abundantly dispersed throughout living systems and are reactive both chemically and physiologically. Biogenic ROS are produced through a variety of enzymatic and nonenzymatic processes involving the mitochondrial respiratory chain, xanthine oxidase, NADPH oxidase, cytochrome P450 enzymes, and so on [4]. It has been widely demonstrated that elevated levels of ROS in vivo is closely related to the onset of a number of diseases, including neurodegenerative disorders, inflammation, cancer, diabetes, etc. [5].

Among the numerous ROS, peroxynitrite (ONOO^−^), characterized by strong oxidation and nitrification capacities, was first identified as a crucial endogenous oxidant in 1990 [6], and since then, has received extensive attention as it believed to serve as an important signaling molecule in a variety of biological pathways [7]. Peroxynitrite is a product of a diffusion-controlled radical coupling reaction between superoxide anion radical (O_2_•^−^) and nitrogen oxide (NO). Under physiological conditions, ONOO− is further transformed into an unstable nitrosoperoxycarbonate intermediate (ONOOCO_2_^−^) through its reaction with carbon dioxide (CO_2_), which in turn undergoes homolytic cleavage to form nitrogen dioxide (^•^NO_2_) and carbonate radical (CO_3_^•−^). The two can either recombine into carbon dioxide and nitrate or act as one-electron oxidants. Because of its high reactivity, exposure to peroxynitrite can either positively or negatively impact cellular viability and function in biochemical cycles, depending on the concentrations of peroxynitrite [8,9]. Despite the fact that peroxynitrite at low concentrations frequently has protective effects on organisms and cells (e.g., the bactericidal effect), abnormal levels of peroxynitrite can cause a disruption in cellular energy generation, which is closely linked to numerous diseases, as ONOO− directly or through its decay products mediates protein oxidation and nitration, lipid peroxidation, mitochondrial dysfunction, and ultimately programmed cell death [7,10]. In addition, peroxynitrite has been discovered to be a direct biomarker of drug-induced acute liver damage because the drugs are subjected to enzymatic biotransformation in the liver to generate peroxynitrite through a series of oxidation processes [11]. Also, the outburst of peroxynitrite has been discovered to be proportionate to the growing progression of inflammation [12]. Thus, developing a direct and/or rapid method that can track the ONOO^−^ in vivo with high sensitivity and selectivity is highly desirable. This would not only enable the better understanding of the important role of ONOO^−^ in biochemistry but also facilitate the early diagnosis and treatment of specific diseases.

Over the past decades, fluorescence-based analysis technology has been acknowledged as a potent in vivo detection method due to its facile visualization, real-time observation, operational simplicity, high sensitivity, super-spatial resolution, and non-invasive nature [13,14]. Up to now, numerous fluorescent probes have been rationally constructed for the detection of metal ions [15], anions [16], ROS [17], viscosity [18], pH [19], specific biomarkers [20], and so on [21]. These fluorescent probes, usually consisting of a fluorophore (reporter of the spectroscopic signals) and a receptor (binding/reaction site toward analytes), as well as a linker between them, can identify analytes through the changes in their emission properties. Not surprisingly, the fluorescent probes for ONOO^−^-specific detection have also been rapidly developed [22]. In terms of the fluorescence reporters, the commonly seen fluorescent scaffolds focus on dicyanomethylene-4-*H*-pyran derivatives [23], boron-dipyrromethene (BODIPY) dyes [24], coumarins [25], benzothiazole backbones [26], 1,8-naphthylimines [27], rhodamines [28], and so on [29,30]. With respect to the receptor, arylboronic acids and their esters are the most common recognition sites for ONOO^−^ [22,23,24,25,26,27,28]. However, despite substantial progress, there is only a limited number of probes that possess both high selectivity and biocompatibility, as well as those that were successfully assessed in animal models. Needless to say, the design and discovery of new fluorophores may provide new opportunities for the construction of novel fluorescent probes with tailored performances capable of addressing the above requirements.

Organoboron complexes are well-known and widely explored organic fluorophores because of their excellent photo- and chemical stability, remarkable structural diversity, ease of accessibility, and narrow absorption and emission bands [31]. N,N-, N,O-, and O,O-chelated boron complexes are among the most typical boron-coordinated organic luminescent compounds. For instance, highly versatile BODIPY dyes, in addition to their chemosensory applications [32], have proved themselves to be fascinating compounds in the labeling of proteins/DNA [33], theranostics [34], organic light-emitting diodes (OLED) [35], and dye-sensitized solar cells (DSSC) [36], as well as numerous smart materials [37,38]. From the point of molecular engineering, all reported organoboron complexes designed for fluorescent probes constitute difluoride boron complexes chelated by bidentate ligands [32]. Surprisingly, no analogous diphenyl boron complexes were employed to construct fluorescent probes for specific analytes.

In the course of synthesizing novel fluorophores with excellent performance [39,40], we found that the N,O-chelated diphenylboron complex (namely DPB herein) could serve as a new fluorescent probe, which showed good sensitivity and selectivity for the recognition of ONOO^−^. The beauty of the probe is that the diphenyl-coordinated boron favors the reinforcement of the π-conjugation and its pinacolphenylboronate moiety acts as a cleavable recognition site for ONOO^−^, producing a strong fluorescence turn-on response. The low cytotoxicity and good biocompatibility allowed the DPB probe to be successfully used for bioimaging ONOO^−^ in living cells and zebrafish. To the best of our knowledge, the DPB probe shown here is the first N,O-chelated diphenylboron-based fluorescent probe for peroxynitrite.

## 2. Materials and Methods

### 2.1. Materials and Instruments

Solvents and reagents were purchased commercially and utilized without additional purification. All other chemicals and solvents used in the studies were analytical grade, with the exception of the solvents (chromatographic grade) used in the spectroscopic measurements. High-resolution mass spectrometry (HR-MS) was recorded on a UHPLC-Q-Exactive Orbitrap Mass Spectrometer (Thermo Fisher Scientific, Waltham, MA, USA). The ultraviolet-visible (UV-vis) absorption spectra and fluorescence spectra were recorded on the TU-1901 spectrophotometer (Persee, Beijing, China) and the F-380 fluorescence spectrophotometer (Gangdong Technology, Tianjing, China), respectively. Cells and zebrafish imaging data were recorded using Leica laser confocal microscope (TSC SP8 STED, Leica, Germany) and fluorescence stereo microscope (M205 FA, Leica, Germany), respectively. Density functional theory (DFT) calculations were conducted using the Gaussian 16 program (the B3LYP/6-311G (d, p) method) [41,42].

### 2.2. Spectral Measurements

The stock solutions of the DPB probe (3.0 mM) were prepared in acetonitrile (CH_3_CN) and the other analytes (30 mM) were prepared in ultrapure water. The samples of DPB in spectral measurement with a variety of analytes including selected metal ions, anions amino acids, and ROS were investigated in PBS-CH_3_CN buffer solution (10 mM, 5:5, *v*/*v*, pH = 7.4). The excitation wavelength in all the fluorescence spectra measurements was set at 405 nm, with a slit width of 10 nm.

### 2.3. Cell Imaging Experiments

Three different cell lines (HCT116, A549, and HepG-2) were first cultured in DMEM medium containing 5% CO_2_ and 10% fetal bovine serum (FBS) at 37 °C. The concentration of the DPB probe in cell imaging experiments was fixed at 10 μM. The cytotoxicity of the DPB probe was assessed by a methyl thiazolyl tetrazolium (MTT) assay. After incubation with the DPB probe for 15 min, the DPB-pretreated cells were further incubated with different concentrations of ONOO^−^ (5.0, 10, and 20 μM). After washing with PBS three times, confocal microimaging was carried out, and LAS X office software (Leica Application Suite X: 3.7.6.25997) was used to examine the corresponding fluorescence intensity. The Fiji software package (lmageJ 1.53t) was further utilized to analyze their mean fluorescence areas.

### 2.4. Imaging of ONOO^−^ in Zebrafish

Zebrafish were cultivated in zebrafish medium after mating and natural spawning. Zebrafish larvae were incubated with DPB (10 μM) for 15 min, and then the DPB-loaded zebrafish larvae were further exposed to different amounts of ONOO^−^ (5.0, 10, and 20 μM). These zebrafish larvae were fixed with 1% low melting point agarose and 0.05% tricaine for imaging. Their images were measured by laser confocal imaging and their corresponding fluorescence intensity was analyzed by LAS X office software.

## 3. Discussion and Results

### 3.1. Design Strategy of DPB Probe

In light of the fact that the family of BODIPY-based dyes has always been a cynosure since the groundbreaking work of Sathyamoorthi and colleagues in the early 1990s [43], it is quite tempting to create other boron chelates with distinct luminescent properties. In general, boron-chelated luminophores can be divided into two categories, three- and four-coordinated boron complexes [44]. The tri-coordinated boron compounds have a vacant coordination site on the boron center because of the existence of an empty p-orbital, which is prone to attack by electron-rich molecules, oxidizing agents and Lewis bases. In sharp contrast, the tetra-coordinated boron compounds have much better rigidity and stability due to the coordinative saturation in the boron center. N,O-chelated diphenylboron complex DPB was initially prepared when the study aimed to assess the possibility of post-functionalization of boron chelates to access derivatives with tunable luminescent properties. Thus, in the current work, the DPB was synthesized according to the reported literature procedure (Figure S1, ESI†) [45]. Considering that DPB contains both three- and four-coordinated boron centers in its backbone, we envisioned that this molecule ought to serve as a fluorescent probe for a specific analyte. We were pleased to discover that the DPB could serve as a selective and sensitive fluorescent probe for ONOO^−^, capable of tracking its presence in living cells and zebrafish (*vide infra*).

### 3.2. Spectral Response Behaviors of DPB Probe to ONOO^−^

**Figure 1:**
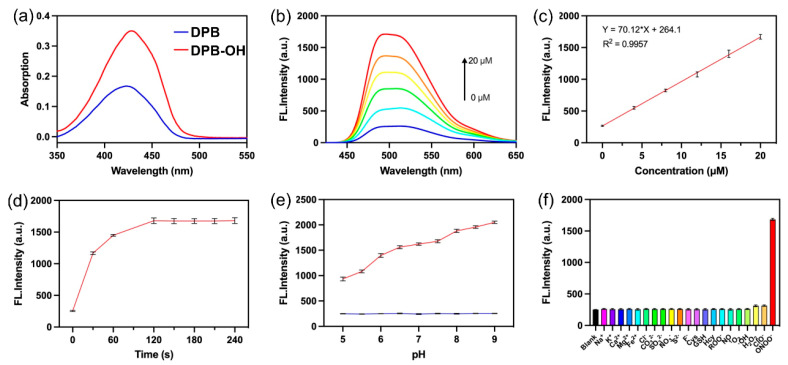
Spectral response behaviors of the DPB probe toward ONOO^−^ in the PBS-CH_3_CN buffer solution (10 mM, 5:5, *v*/*v*, pH = 7.4). (**a**) UV-vis and (**b**) fluorescence spectrums of DPB in the absence and presence of ONOO^−^ (0–50 μM) (λ_ex_ = 405 nm). (**c**) Plots of the linear relationship between the fluorescence intensities at 500 nm and the concentration of ONOO^−^. (**d**) Time- and (**e**) pH-dependent fluorescence intensity of DPB at 500 nm after the treatment of ONOO^−^ (20 μM). (**f**) Fluorescence intensity of DPB at 500 nm in the presence of several interference analytes (1.0 mM).

With the compound DPB in hand, we can hardly wait to verify whether this molecule is able to recognize the analytes with oxidative properties. Therefore, the spectral response behaviors of the DPB probe toward ONOO^−^ were initially examined. In the UV-vis absorption spectroscopy, the DPB (10 μM) exhibited a maximum absorption peak at 428 nm in the PBS-CH_3_CN buffer solution (10 mM, 5:5, *v*/*v*, pH = 7.4) (Figure 1a). The addition of ONOO^−^ not only caused a slight bathochromic shift in its maximum absorption wavelength, but also induced a significant increase in its absorption intensity, preliminarily suggesting that DPB possessed the recognition ability for ONOO^−^. The recognition properties of DPB were then tested using fluorescence spectroscopy in the identical media. As shown in Figure 1b, the DPB probe displayed almost no fluorescence in this mixed solvent system, and an apparent fluorescence emission peaked at 500 nm, progressively increasing with the gradual addition of ONOO^−^. In particular, the fluorescence intensity of DPB at 500 nm could reach its maximum value when 2.0 equivalents of ONOO^−^ were added (Figure S2, ESI†). At the same time, a satisfactory linear relationship (R^2^ = 0.9957) between the fluorescence intensities at 500 nm and concentrations of ONOO^−^ in the range of (0–20 μM) could be achieved (Figure 1c), from which the detection limit of DPB for ONOO^−^ could be determined to be 55 nM using the 3σ/k method [46].

Afterward, time-dependent fluorescence tests were also conducted to assess the behavior of ONOO^−^ recognition. When 2.0 equivalents of ONOO^−^ (20 μM) was added to the DPB probe (10 μM), the emission intensity at 500 nm was gradually enhanced, reaching the maximum within 2.0 min (Figure 1d), indicating that the DPB probe showed a fast turn-on fluorescence recognition of ONOO^−^. Furthermore, the ONOO^−^-recognition behaviors of DPB at various pH levels were then investigated. As shown in Figure 1e, the fluorescence emission of the DPB probe did not alter significantly in the absence of ONOO^−^ at all the pH ranges (pH = 5.0–9.0). Following the treatment with ONOO^−^ (20 μM), the emission intensity at 500 nm increased progressively along with an increase in pH values. In other words, the DPB probe was capable of turn-on fluorescence recognition of ONOO^−^ under physiological pH conditions.

In light of the fact that the anti-interference capability is an important parameter of a probe, the selective sensing ability of the DPB probe toward ONOO^−^ was assessed by conducting competing experiments with selected metal ions (K^+^, Na^+^, Ca^2+^, Mg^2+^, and Fe^2+^), anions (Cl^−^, CO_3_^2−^, SO_4_^2−^, NO_3_^−^, S^2−^, and F^−^), thiol amino acids (Cys, Hcy, and GSH), and reactive oxygen species (ROO•, NO, ^1^O_2_, •OH, H_2_O_2_, and CIO^−^) as potential interferents. As illustrated in Figure 1f, the addition of the above-mentioned species, even at the concentration of 1.0 mM (100 equivalents), did not cause notable emission changes, in most instances producing an emission identical to the blank sample (the DPB probe itself). Only ONOO^−^ could light up the fluorescence emission centered at 500 nm. These results implied that DPB probe exhibited excellent selectivity for ONOO^−^ recognition without any significant interference from other biological analytes (particularly other ROS).

### 3.3. Mechanism of Recognition of ONOO^−^ by DPB

**Scheme 1:**
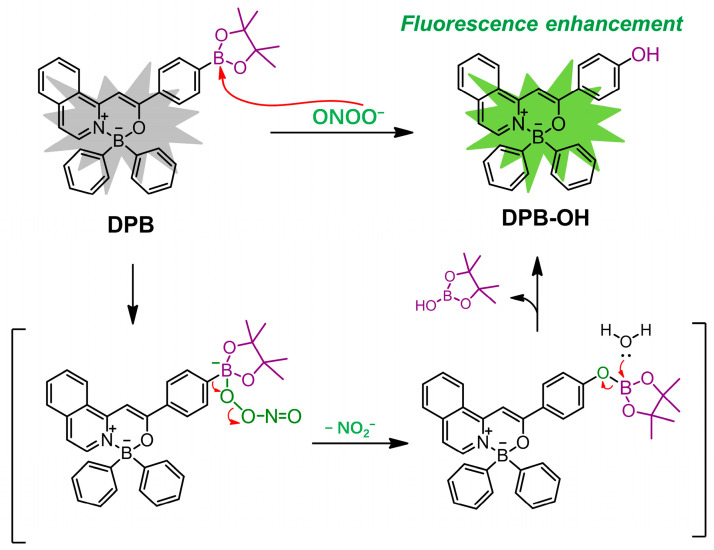
Proposed recognition mechanism of DPB probe for ONOO^−^.

In view of the remarkable ONOO^−^-induced emission enhancement achieved with DPB, we propose that the ONOO^−^-recognition mechanism was attributed to an oxidative cleavage of three-coordinated pinacolphenylboronate moiety to form a hydroxyl group (Scheme 1). The presence of a bulky pinacolphenylboronate pendant in DPB results in the dissipation of a significant amount of excitation energy through non-radiative decay. Thus, the oxidative replacement of pinacolphenylboronate with a compact hydroxyl group contributed to a markedly strong fluorescence enhancement in the resultant DPB-OH derivative.

The first indication for the formation of DPB-OH came from high-resolution mass spectrometry (HR-MS). The HR-MS spectrum, recorded in a negative ionization mode for the sample upon the addition of ONOO^−^ to DPB_,_ revealed a strong *m*/*z* peak at 426.1671 (Figure S3, ESI†), which could be assigned to the presence of the oxidized DPB-OH form (calculated [M-H]^−^ = 426.1670), suggesting that the pinacolphenylboronate unit was converted to a hydroxyl group. Furthermore, DPB-OH, the corresponding product of oxidative cleavage, was successfully isolated and its structure was confirmed by ^1^H NMR spectroscopy (Figure S4, ESI†).

**Figure 2:**
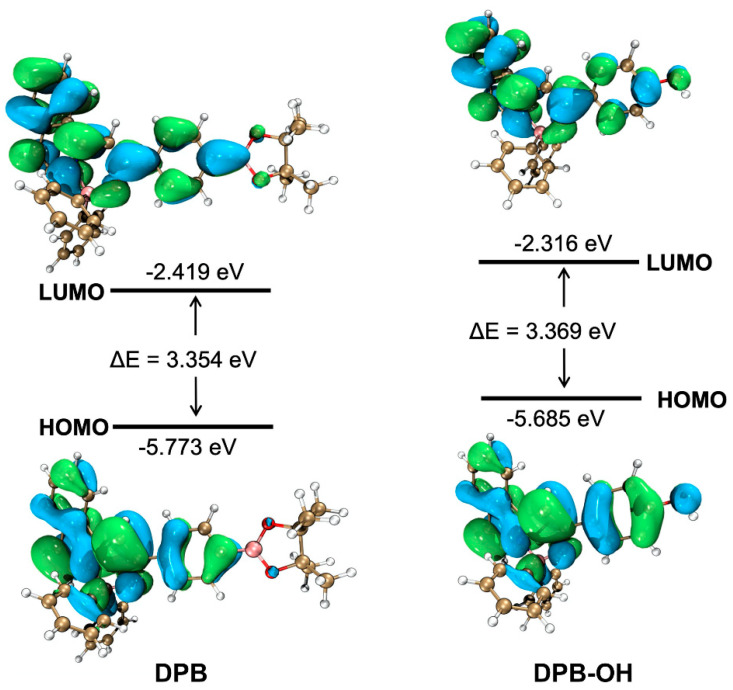
Density functional theory calculations for DPB and DPB-OH.

Density functional theory (DFT) calculations were employed to optimize the geometry of both DPB and DPB-OH, and to compute their corresponding highest occupied molecular orbitals (HOMO) and lowest unoccupied molecular orbitals (LUMO) (Figure 2). The HOMO-LUMO gap of DPB-OH (ΔE = 3.369 eV), which contains a phenolic hydroxyl group in place of a pinacolphenylboronate group, was higher than that of DPB (ΔE = 3.354 eV), explaining a slight bathochromic shift in the maximum absorption observed upon treatment of DPB with ONOO^−^.

The presence of an electron-donating hydroxyl group may induce an intramolecular charge transfer (ICT) effect on DPB-OH that could also account for the observed fluorescence enhancement during the ONOO^−^ recognition process. This corroborates with an increase in fluorescence intensity observed for DPB-OH when switching from an acidic to a basic medium (Figure 1e). The basic conditions are conducive to the deprotonation of phenolic hydroxyl groups, which is beneficial to the enhancement of ICT effect. At the same time, a weaker fluorescence in an acidic medium might also be ascribed to a partial degradation of DPB-OH via boron decomplexation.

### 3.4. Bioimaging in Living Cells

**Figure 3:**
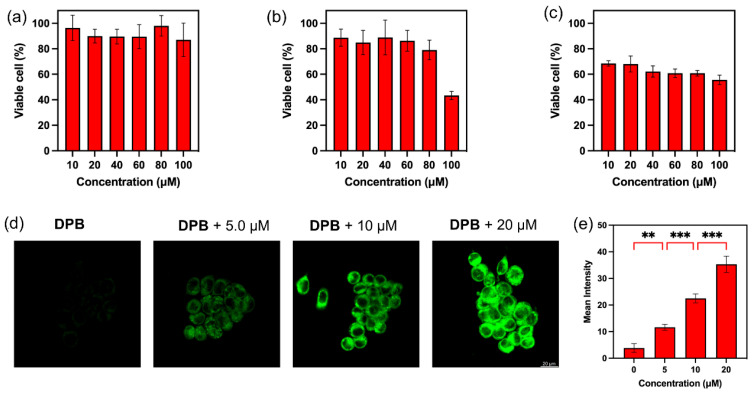
Cytotoxicity assay of different concentrations of DPB on (**a**) HCT116, (**b**) A549, and (**c**) HepG-2. (**d**) Colocalization imaging of ONOO^−^ in HCT116 cells with the DPB probe. After pretreatment with the DPB probe (10 μM) for 15 min, the HCT116 cells were incubated with different concentrations of ONOO^−^ for 30 min. (**e**) Statistical results of the corresponding fluorescence intensity of (**d**). *p*-values are calculated using one-way ANOVA with Bonferroni correction, ** *p* < 0.01, *** *p* < 0.001. Scale bar is 20 μm.

The obtained favorable recognition behaviors of DPB for ONOO^−^ in vitro prompted us to further explore whether our molecule is suitable for bioimaging in vivo. Prior to bioimaging in living cells, a 3-(4,5-dimethylthiazol-2-yl)-2,5-diphenyl tetrazolium bromide (MTT) assay experiment, a widely used method for evaluating a compound’s toxicity, was conducted to ensure that DPB is a safe probe to use in cells. Three different cell lines of human colon cancer cells (HCT116), human lung cancer cells (A549), and human hepatocellular carcinoma cells (HepG-2) were chosen to measure the cytotoxic activities of the DPB probe. After a 24 h incubation with DPB at different concentrations (10–100 μM), despite moderate survival rates for HepG-2 cells (ca. 55–70%) under all test concentrations, the cell viabilities of both HCT116 and A549 cells exceeded 90% even at concentrations of DPB higher than 10 μM (Figure 3a–c), indicating that the DPB probe exhibits negligible or quite low cytotoxicity.

Subsequently, HCT116 cells were selected in the cell imaging experiments. When the HCT116 cells were incubated with the DPB probe (10 μM) for 30 min, the internal fluorescence of the cells was barely noticeable. In contrast, when these cells were further incubated with various concentrations of ONOO^−^ (5.0, 10, and 20 μM), the DPB-loaded cells displayed a significant fluorescence amplification, and detectable fluorescence signals in the green channel could be observed (Figure 3d). In particular, the enhanced fluorescence intensity showed an ONOO^−^-dependent concentration (Figure 3e). The above results imply that the DPB probe has good cell membrane permeability and is capable of imaging intracellular ONOO^−^ in living cells.

### 3.5. Bioimaging of ONOO^−^ in Zebrafish

**Figure 4:**
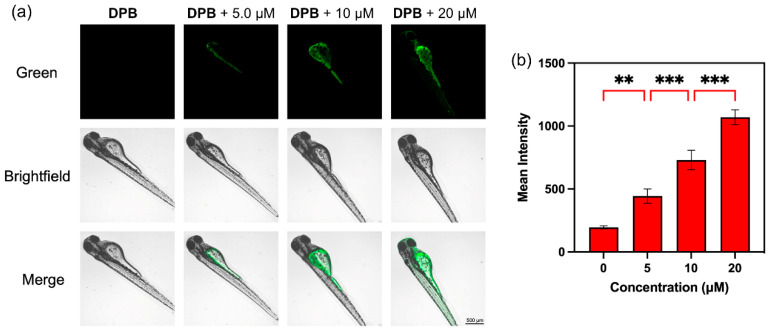
(**a**) Fluorescence imaging of ONOO^−^ with DPB (10 μM) in zebrafish (After pretreatment with DPB for 15 min, the zebrafish were further incubated with different concentrations of ONOO^−^ for 30 min). (**b**) Statistical results of the corresponding fluorescence intensity of (**a**). *p*-values are calculated using one-way ANOVA with Bonferroni correction, ** *p* < 0.01, *** *p* < 0.001. Scale bar is 500 μm.

The success in the imaging of ONOO^−^ in cells urged us to explore whether the DPB probe is a viable tool for tracking ONOO^−^ in an animal model. Zebrafish are known to be an important vertebrate model for simulating human genetic diseases because of their distinct merits, which include ease of breeding, high human homology, quick growth, facile operation, and especially translucent characteristic. Thus, monitoring ONOO^−^ in zebrafish was then carried out. Three-day-old zebrafish were first treated with DPB (10 μM) for 15 min, followed by further incubation with ONOO^−^ for another 30 min. After being washed with PBS, these zebrafish were placed onto a brand-new confocal plate for imaging. As depicted in Figure 4, the zebrafish labeled only with the DPB probe (10 μM) showed an extremely low fluorescence. Nevertheless, the DPB-labeled zebrafish had a noticeably enhanced fluorescence in the green channel after the addition of ONOO^−^ (Figure 4a), with the fluorescent intensity increasing with increasing the amount of ONOO^−^ (Figure 4b). These results demonstrate that the DPB probe can effectively monitor ONOO^−^ in a zebrafish model.

## 4. Conclusions

In summary, we reported a new fluorescent probe, DPB, whose skeleton contained both a four-coordinated fluorogenic diphenylboron unit and the three-coordinated pinacolphenylboronate moiety responsible for the recognition of ONOO^−^. Probe DPB was characterized by good selectivity, fast response time (2.0 min), and low detection limit (55 nM), representing the first N,O-chelated diphenylboron-based fluorescent probe for ONOO^−^. The ONOO^−^-recognition mechanism involves ONOO^−^-induced oxidative cleavage of the pinacolphenylboronate fragment leaving stable four-coordinated diphenylboron moiety intact. This results in the transformation of DPB into a strong fluorescent molecule DPB-OH, whose emission is enhanced due to the substantial deactivation of non-radiative decay. The favorable biocompatibility and negligible cytotoxicity allowed the DPB probe to be successfully used to track ONOO^−^ in living cells and zebrafish, proving itself as a promising candidate for the further investigation of ONOO^−^-related pathological and physiological processes.

## Data Availability

The original contributions presented in the study are included in the article and Supplementary Materials, further inquiries can be directed to the corresponding author.

## Supplementary Materials

The following supporting information can be downloaded at: https://www.mdpi.com/article/10.3390/bios14110515/s1. Figure S1: ^1^H NMR spectrum (600 MHz, CDCl_3_) of DPB at 298 K; Figure S2: Fluorescence intensity of DPB (10 μM) at 500 nm with various concentrations of ONOO^−^; Figure S3: HR-MS spectrum of DPB-OH; Figure S4: ^1^H NMR spectrum (600 MHz, CDCl_3_) of DPB-OH at 298 K.
