# Supplementary material for: The First N,O-Chelated Diphenylboron-Based Fluorescent Probe for Peroxynitrite and Its Bioimaging Applications
Source: Biosensors (Basel). 2024 Oct 22;14(11):515. doi: 10.3390/bios14110515 (PMC11592091; doi:10.3390/bios14110515)
Supplement: Supplementary file 1 [file biosensors-14-00515-s001.zip › biosensors-3244786-supplementary.pdf]

The first N,O-chelated diphenylboron-based fluorescent probe for peroxynitrite and its bioimaging applications

Xiaoping Ye [a], Longxuan Li [b], Hong Liu [c], Yuyu Fang [b], Xiaoya Liu [d]*

[a] *Department of Ultrasound, the First Affiliated Hospital of Chongqing Medical University, Chongqing 400016, China*

[b] *School of Pharmacy, Chengdu University of Traditional Chinese Medicine, Chengdu 611137, China*

[c] *Department of Vascular Surgery, the First Affiliated Hospital of Chongqing Medical University, Chongqing 400016, China*

[d] *Department of Oncology, the First Affiliated Hospital of Chongqing Medical University, Chongqing 400016, China*

Corresponding author, e-mail: liuxiaoya@cqmu.edu.cn

Supporting Information

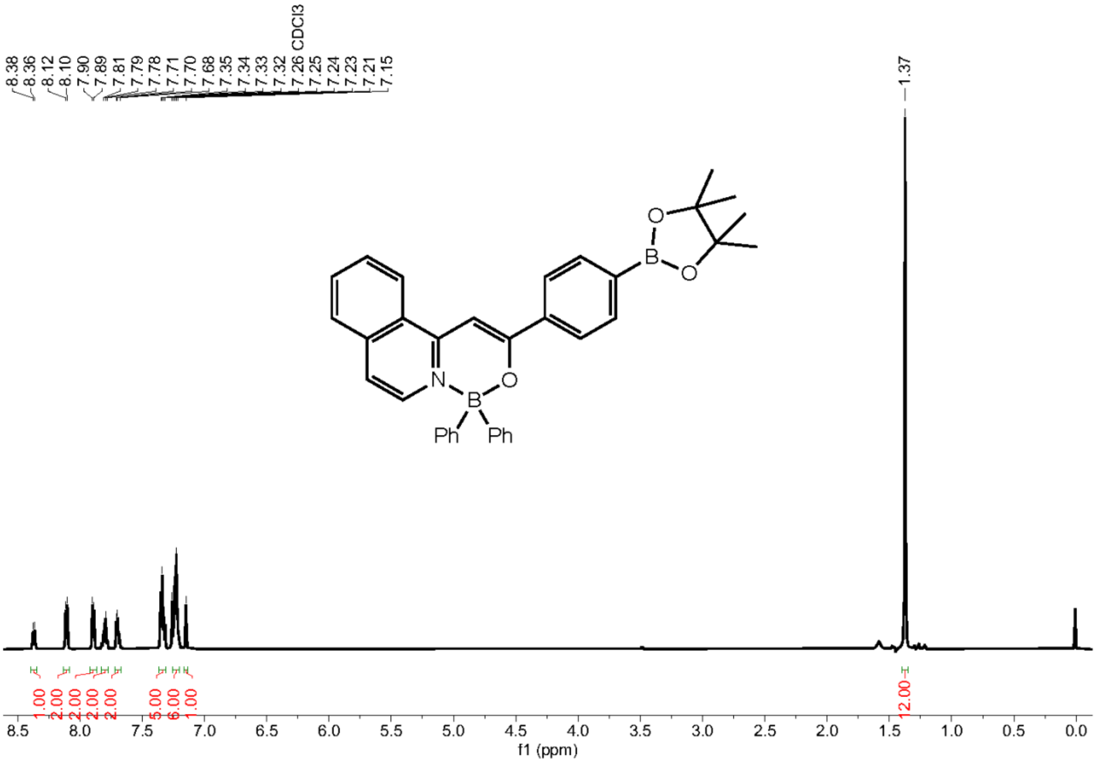

Figure S1. ^{1}H NMR spectrum (600 MHz, $CDCl_3$) of **DPB** at 298 K.

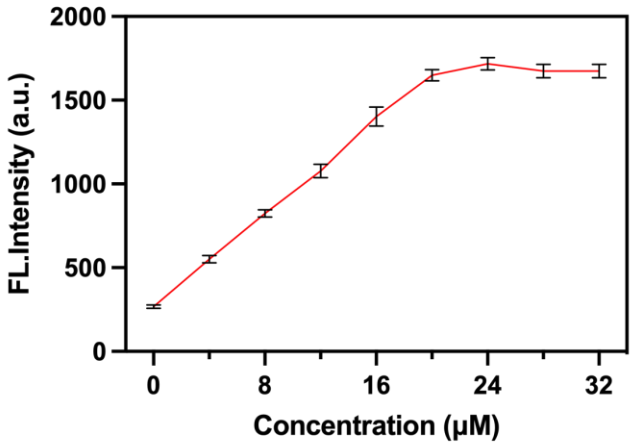

Figure S2. Fluorescence intensity of **DPB** (10 μM) at 500 nm with various concentrations of $ONOO^-$.

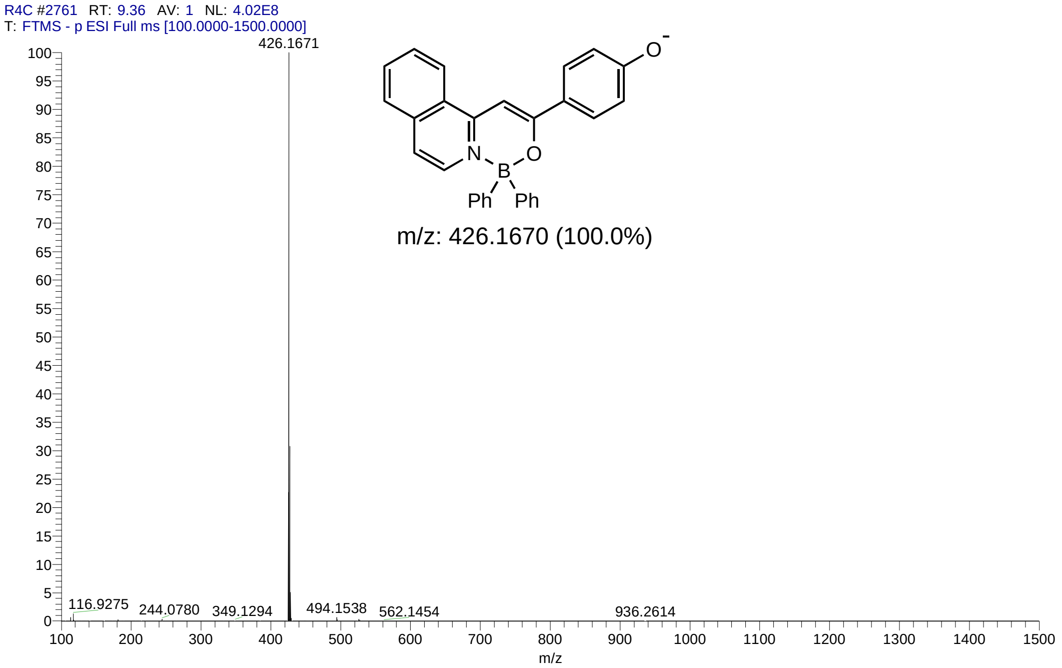

Figure S3. HR-MS spectrum of **DPB-OH**.

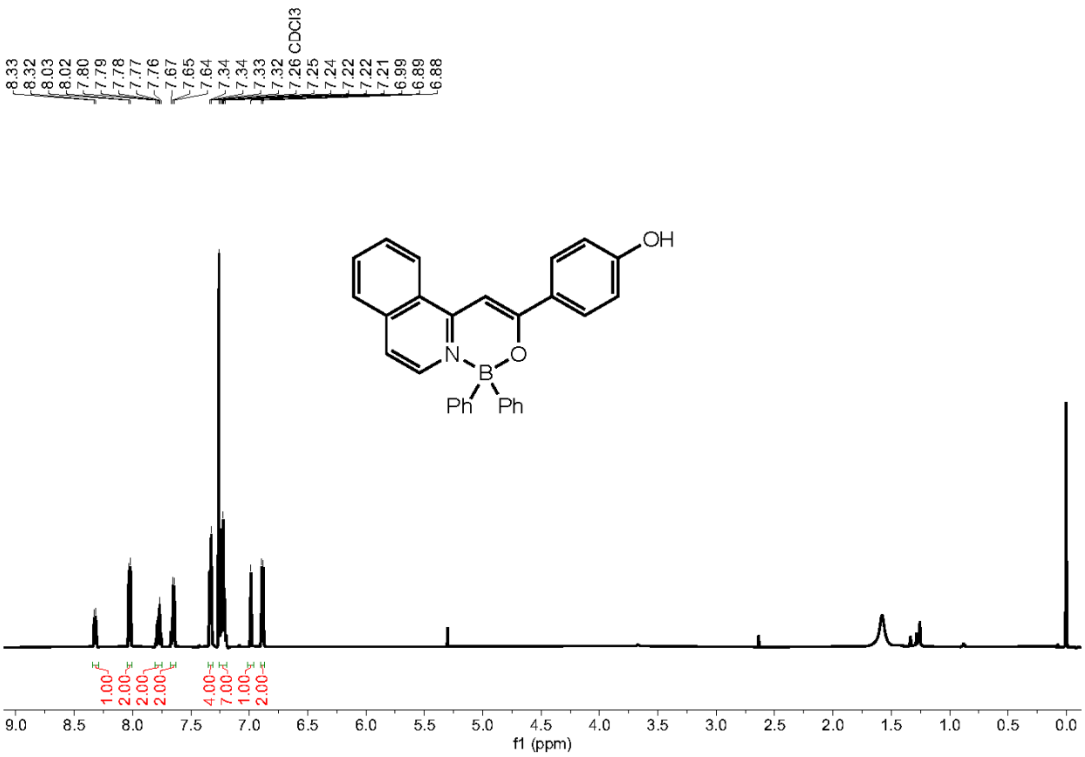

Figure S4. ^{1}H NMR spectrum (600 MHz, $CDCl_3$) of **DPB-OH** at 298 K.
